# Medicolegal and ethical concerns in genetic tests communication

**DOI:** 10.3389/fsoc.2026.1876006

**Published:** 2026-07-22

**Authors:** Pierpaolo Di Lorenzo, Mariagrazia Marisei, Marco Macculi, Claudia Casella, Pascale Basilicata, Emanuele Capasso, Massimo Niola

**Affiliations:** Department of Advanced Biomedical Sciences, University of Naples Federico II, Naples, Italy

**Keywords:** communication, genetic counseling, genetic tests, personal data, privacy, solidaristic use

## Abstract

**Objective:**

Genetic information is a special category of personal data that is relevant for its predictive reach. The purpose of this paper is to provide an overview of the state of the art on the communication of genetic test results in both clinical and judicial practice in Europe.

**Methods:**

Starting from the research problem, legislation, policy documents and case law have been examined in order to combine legal, ethical, judicial and clinical aspects that shape the field of genetic test communication in Europe, with a specific focus on Italy.

**Results:**

Data suggesting the state-of the art of genetic results communication in clinical practice has been analysed and organized in thematic sections.

**Conclusion:**

A focus on consent and confidentiality is indicated by the numerous works that have emerged from the human genome project and the general ideas around predictive medicine. The real challenge consists in transforming the potential of genetic data from a personal, ultrapersonal dimension to a solidaristic one, which lies beyond the horizon of personalized and predictive medicine and uncovers a new dimension of universality. Therefore, health professionals’ *éxpertise* in genetic test communication plays a key role in modern potentials of the information retained within tests.

## 1 Introduction

Genetic data are a special category of personal data based on structural sharing with an intrinsic accompanying predictive attitude. Typically, genetic data have interpersonal value, since genetic information transcend the sphere of the person who has undergone the test and are also capable of involving the members of the so-called “genetic family” to which the former belongs. The potentialities of access and the relatedness of access to specific genetic information are one the most relevant aspects to be discussed in informed consent process. According to the Italian Code of Medical Ethics (Federazione Nazionale degli Ordini dei Medici Chirurghi e degli Odontoiatri, 2014), for example, the physician must maintain secrecy about everything he or she knows by reason of his or her professional activity. In Italy, genetic investigations are performed by physician only with the written consent of the person concerned, who is the only recipient of the relevant information. About that, there are some differences among the countries, such as in the United States of America, where genetic tests can be ordered by other healthcare providers, like nurses and genetic counselors. The processing of “genetic,” “biometric” and “health-related” data (legislation.gov.uk, 2016) requires prior information process (given in a written form and mediated using understandable and simple language, possibly even considering the patients’ level of education) on the characteristics of the processing data and patient’s rights. “Genetic test” is defined as the analysis for clinical purposes of a specific gene or its product or function or other parts of DNA or a chromosome (legislation.gov.uk, 2016). Tests for clinical purposes are usually divided into: (a) diagnostic testing: to make a diagnosis or confirm a clinical suspicion in an affected individual; (b) presymptomatic testing: to detect or rule out the presence of a pathogenic variant associated with a genetic disease that may develop in an unaffected individual; (c) predictive or susceptibility testing: to assess an individual’s greater or lesser susceptibility to develop multifactorial diseases. The use of cancer-related genetic and genomic testing (CGT) for the prevention, diagnosis, and treatment of cancer is expected to revolutionize cancer care, leading to an era of precision medicine (Peterson et al., 2018). Genetic counseling and germline testing for pathogenic variants in known cancer susceptibility genes is an important component of preventive medicine (Hamilton et al., 2017), guides timing and nature of therapeutics, and it’s useful as a starting point for “cascade” testing, in which blood relatives are tested for a known genetic mutation (Chou et al., 2025). Moreover, the next-generation sequencing (NGS) techniques, used to interrogate and analyze the cancer genome, have the promise of personalizing treatment for patients with cancer (Capasso et al., 2024; Lolkema et al., 2013). Indeed, NGS brought new insight into the biology of head and neck squamous cell carcinoma (HNSCC) and revealed its’ high genetic complexity. It is estimated that in the next decades, precise genetic profiling of pathogenic variant in tumors as well as a wide range of targeted anti-tumor drugs will become available (Giefing et al., 2016).

Some ethical and medical-legal questions specifically raised by predictive medicine concern how to inform the patient, the extension of information, the relative obligation to inform, and who the recipients of the information are. The purpose of this paper is to provide an up-to-date overview of the state of the art on information management regarding genetic data, privacy protection, current legal standards, and ethical/ethical rules to be observed, especially in the European area where GDPR provisions apply (legislation.gov.uk, 2016).

## 2 Materials and methods

This study was conducted using an interdisciplinary approach, integrating bioethical, medico-legal, and legal perspectives, with the objective of analysing the communication of genetic test results in clinical settings across Europe, with a specific focus on the Italian context. All documents addressing communication procedures for genetic results in clinical settings, combined with legal texts or judicial proceedings, were considered eligible for inclusion. Both international and national materials were included, encompassing scientific literature, jurisprudential sources, policy documents, clinical guidelines, European Regulations, Italian statutory law, the General Data Protection Regulation (GDPR), and international Ethical Declarations. These documents were identified through targeted searches on official institutional websites and publicly accessible internet search engines. No time limit was applied to the document search, which includes all documents up to 2025. To verify the “state of the art” in scientific literature, PubMed was consulted using the following terms: genetic [Title/Abstract] AND communication [Title/Abstract]. Only articles in English and Italian were included. Sources were selected based on their direct relevance to the clinical, ethical, and legal dimensions of genetic test communication. Two reviewers conducted the search separately, achieving the same results. Retrieved data were systematically analysed and organised into thematic sections regarding the most important aspects of communication process of genetic results in clinical settings: duty to inform, how to inform, who to inform and quantity of information.

## 3 Results

The division into thematic sections of this study was designed to focus on the different aspects of the process, in particular methods of communication, the type of information to be provided, the related obligations, the parties involved, as well as the clinical and legal implications (see Table 1).

**Table 1:** Synoptic table on information characteristics and normative/disciplinary references.

Characteristics of genetic communication	Legislative references
Duty to inform	Law 219/17. Code of medical ethics. Council of Europe (1988). Protocol to the Convention for the Protection of Human Rights and Fundamental Freedoms (European Convention on Human Rights) as amended by Protocol No. 11. In Council of Europe Treaty Series 155. Council of Europe. The Universal Declaration on the Human Genome and Human Rights (1997), http://www.unesco.org/general/eng/legal/index.shtml
How to inform	Council of Europe (1988). Protocol to the Convention for the Protection of Human Rights and Fundamental Freedoms (European Convention on Human Rights) as amended by Protocol No. 11. In Council of Europe Treaty Series 155. Council of Europe. Code of medical ethics. European Union, Charter of Fundamental Rights of the European Union, 2012/C 326/02, 14 December 2007.
Who to inform	Law 219/17. Code of medical ethics. Opinion on the management of “incidental findings” in genomic investigations with new technology platforms, National Bioethics Committee, March 17, 2016.
Quantity of information	Law 219/17. Code of medical ethics. Provision containing the requirements for the processing of special categories of data, pursuant to Article 21, Paragraph 1 of Legislative Decree No. 101 of August 10, 2018.

### 3.1 How to carry out a proper communication process

During the establishment of the Human Genome Project (HGP), questions were raised regarding the potential use of the new genetic data and the safeguards for people and society. The Ethical, Legal, and Social Implications of Human Genetics Research (ELSI) program was created to investigate these concerns and support the creation of standards and policy proposals to guarantee the proper use of genetic information (Ascencio-Carbajal et al., 2021).

Multidisciplinary ELSI research is supported by the Human Genome Project. New concepts on the horizon include genomic sovereignty, data sharing, and international collaboration. ELSI criteria can also be applied to other genomic fields like biobanks, genetics, and epigenetics. Autonomous regulation is particularly urgent since genomic medicine offers lucrative technological applications.

Other documents, like The Nuremberg Code (1946), World Medical Association (1964), United States National Commission for the Protection of Human Subjects of Biomedical and Behavioral Research (1978), World Medical Association (1981), Nufeld Council on Bioethics (1993), World Health Organization. Advisory Committee on Health Research (2002), and United Nations Educational Scientific and Cultural Organization (2005), served as precedents because they also addressed bioethical issues related to human genetics. The analysis of these documents highlights the international community’s growing interest in the ethical concerns and implications of the genetic communication process.

Professional secrecy in health care is related to the position of the sick person who finds himself in the need to disclose personal facts about which in other circumstances he/she would have preferred to remain silent. Correct information must be provided and rendered in such a way as to prevent the person predisposed to the disease from ending up feeling predestined to it, with intuitable repercussions on his or her psyche; moreover, in cancer genetic personalized medicine, it could serve as an element of hope with regard to the potential benefits in response to therapy. Nevertheless, carrying a pathogenic variant that may lead to poor prognosis, specifically in oncological field, may result in severe consequences on patient’s life. Adequate preparation of the health care provider also in interpersonal communication sciences and a suitable Genetic Counselling Service, equipped with technical-scientific, ethical and psychological expertise, for the benefit of test subjects are deemed necessary. The code of medical ethics stipulates that the physician shall adapt communication to the capacity of understanding of the person assisted or his or her legal representative, corresponding to any request for clarification, considering the relevance and emotional reactivity of the same, particularly in the case of serious or inauspicious prognoses, without excluding elements of hope. The result of a genetic test opens to a whole range of considerations that may concern, for example, the clinical non significance of a genetic condition, as well as an uncertainty about the onset of a given disease or the more or less relevant response to a given drug treatment on the basis of the results derived from the presence of biomarkers or immunohistochemical investigations.

### 3.2 Quantity of information

According to Law 22 December 2017, no. 219 (2018) titled - Rules on Informed Consent and Advance Treatment Provisions - “every person has the right to know his or her health condition and to be informed in a manner that is complete, up-to-date and understandable to him or her. The information provided must be comprehensive, specific, and intelligible.” The Italian Code of Medical Ethics also echoes this legal requirement by specifying that the physician shall ensure that the person being cared for or his or her legal representative is provided with comprehensible and exhaustive information. About the predictive investigations, it adds that: “The physician shall inform the person concerned about the meaning and purpose of the investigation, the actual likelihood of reliable prediction, the feasibility of available and effective therapeutic interventions, and the possibility of negative consequences on the quality of life resulting from knowledge of the results.” The provision of the Guarantor authority for the protection of genetic data in Italy (Italian Data Protection Authority, 2018) provides that the person should be informed of the results that can be achieved, including in relation to unexpected news that may become known as a result of the processing of genetic data. In addition to this, the person has the right or not to limit the scope of communication of genetic data and the transfer of biological samples, as well as the possible use of such data for further purposes.

### 3.3 Obligation to inform and data retention

Genetic information, like any other information in health, is a genuine subjective right, which is held by its recipient. This right is not inalienable and can be the subject of an abdicative act so that that its holder may well renounce it. According to Law 22 December 2017, no. 219 (2018) the patient “may refuse in whole or in part to receive the information.” The article 33 of the Code of Medical Ethics provides that “the physician shall respect the necessary confidentiality of information and the will of the patient not to be informed.” Similarly, the European Convention on Human Rights and Biomedicine (Council of Europe, 1988) states that every person has the right to know any information collected about his or her health. However, a person’s wish not to be informed must be respected. The Universal Declaration on the Human Genome and Human Rights (UNESCO, 1997) provides “the right of everyone to decide whether or not to be informed of the results of a genetic test and its consequences should be respected.”

With regard to data retention, General Data Protection Regulation (GDPR) does not define a specific retention period for genetic data (legislation.gov.uk, 2016), so there are some differences between European countries. In Germany, genetic test results are retained for 30 years in accordance with Article 17 of the Genetic Diagnosis Act (GenDG) (Schneider and Schneider, 2024), in Spain there is not a specific law establishing a unique retention period while in Italy they are retained indefinitely.

### 3.4 Who to inform

Genetic information must be transmitted to the owner of the sensitive data (patient). The Oviedo Convention further states that “Everyone has the right to respect for his private life when it comes to information concerning his health.” So does the Charter of Fundamental Rights of the European Union (2007). Predictive investigations are separately addressed in the Code of Medical Ethics (Federazione Nazionale degli Ordini dei Medici Chirurghi e degli Odontoiatri, 2014): “A physician shall prescribe or perform predictive investigations with the written consent of the data subject or his or her legal representative, who are the sole recipients of the data and related information.”

Genetic data must be disclosed to the patient by health professionals and health care organizations only through a physician designated by the data subject or the data controller (Italian Data Protection Authority, 2018).

Genetic information must be passed on to those who, as genetically related to the former, could obtain valuable information about their health prospects from the acquisition of such information, resulting in appropriate personal and health decisions. According to Law 219.17, “the person may refuse in whole or in part to receive the information or designate family members or a person he or she trusts to receive it and to give consent on his or her behalf if the patient so wishes.”

The National Bioethics Committee (2016) also commented on this issue in 2016, indicating that “test takers must be made aware that the analysis’s findings may have major implications for their family members and that, in some situations (such as when serious diseases are discovered and preventive or therapeutic measures must be taken right away) it is appropriate to dutifully allow the latter to learn about them with the necessary vigilance and modalities.” The Code of Medical Ethics states that information to third parties may be provided with the explicit consent of the person being cared for. The Italian Data Protection Authority (2018) establishes that “the results of genetic testing and screening, if they result in a concrete and direct benefit in terms of therapy, prevention, or awareness of reproductive choices, including for those belonging to the same genetic lineage as the data subject, may be communicated to the data subjects at their request, if the person concerned has expressly consented to it or if such results are essential to avoid harm to their health, including reproductive risk, and the consent of the person concerned is not given or cannot be given due to actual unavailability.” If a third party shares the same genetic heritage as the data subject and the data subject gives permission, genetic data may be processed for the third party’s health protection. In the event that the data subject’s consent is not given or cannot be given because of physical impossibility, incapacity to act, or mental incapacity, as well as actual unavailability, data processing may be restricted to the available genetic data, if it is necessary to help the third party make an informed reproductive decision or if it is justified by the third party’s need for therapeutic or preventive interventions. Genetic data extrapolated from the analysis of the deceased person’s biological samples may also be processed in cases where the data subject died, if doing so is necessary to help the third party make an informed reproductive decision or is warranted by the third party’s need for therapeutic or preventive interventions.

The right to access information regarding susceptibility to pathological events, sometimes of considerable severity, cannot be confined exclusively to the individual to whom it is directly and immediately referable, but must also be extended to those who, as genetically related to the former, could derive valuable information about their health prospects from the acquisition of such information, with the consequent possibility of making appropriate personal and health-related decisions relevant to both quality of life and future reproductive choices. For individuals outside the genetic family no is provided for the dissemination of genetic test results.

### 3.5 The relationship between individual variability testing and health

Individual variability tests are divided into:

- Parent relationship test: aimed at establishing kinship relationships;
- Ancestral test: aimed at establishing a person’s relationships with an ancestor or to a particular population or how much of his or her genome was inherited from ancestors belonging to a particular geographical area or ethnic group;
- Genetic identification test: aimed at determining the probability with which a sample or trace of DNA recovered from an object or other material belongs to a person (Italian Data Protection Authority, 2018).

Except for sex, no information regarding the health state or other attributes of the data subjects may be gathered during processing that uses individual variability testing. It is prohibited to use processed genetic data and biological samples obtained for individual variability testing for defensive investigations or to exercise a right in criminal proceedings for any other reason. Defensive investigations or the exercise of a right in criminal proceedings may make use of processed data and biological samples obtained for genetic testing for the purpose of prevention, diagnosis, or treatment against the individual in question, or for scientific and statistical research, in compliance with the applicable legal conditions.

### 3.6 Defensive and judicial investigation

Processing for the purpose of conducting defensive investigations (Italian Data Protection Authority, 2016) or for the exercise of a right in a court of law may be carried out by means of genetic testing only after informing the subject about:

- the analytical explication of all the specific purposes pursued;
- the results achievable also in relation to unexpected news that may become known as a result of the processing of genetic data;
- the data subject’s right to object to the processing of genetic data for legitimate reasons;
- the right or not of the data subject to limit the scope of communication of genetic data and the transfer of biological samples, as well as the possible use of these for further purposes;
- the retention period of genetic data and biological samples.

In cases where the individual variability test is intended to ascertain paternity or maternity people are, in addition, informed about the legislation on filiation, highlighting the possible psychological and social consequences of the test.

## 4 Discussion and conclusion

### 4.1 Discussion

There was complete modification of management of genetic data collection after 2018. In fact, before 2018 genetic data might be processed and biological samples used only for the purposes indicated in this authorization and with respect to which the person has given prior and written informed consent. Consent remained valid only if the person concerned was free from any conditioning or coercion and remained freely revocable at any time. Data processing related to the performance of genetic tests for the conduct of defensive investigations or for the exercise of a right in a court of law might be carried out only with the informed consent of the person to whom the biological material necessary for the investigation belonged, unless an express provision of the law, or an order of the judicial authority in accordance with the law, provided otherwise (Italian Data Protection Authority, 2016). After 2018, consent to the processing of genetic data is required for the conduct of genetic testing as part of defensive investigations or for the exercise of a right in Court, unless an express provision of law, or an order of a judicial authority in accordance with the law, provides otherwise. The processing of genetic data for defensive investigations under Law 7 December 2000, No. 397 (2001), including by authorized private investigators, technical consultants, and substitutes, or, in any event, to assert or defend a right even by a third party in court, must be carried out with the consent of the individual in question only if it requires the administration of genetic tests. Informed consent is required from the person to whom the biological material necessary for the investigation belongs, unless an express provision of the law, or an order of the judicial authority in accordance with the law, provides otherwise. This, provided that the right to be asserted or defended is of equal rank with that of the person concerned, that is consisting of a right of personality or another fundamental and inviolable right or freedom, and the data are processed exclusively for such purposes and for the period strictly necessary for their pursuit (Italian Data Protection Authority, 2018).

Consent to the processing of genetic data is required for treatments carried out by genetic testing, including screening, for research or family reunification purposes. In these situations, if the results of the examination or research represent a tangible and direct benefit for the individual in question in terms of therapy, prevention, or awareness of reproductive choices, the individual must indicate whether or not he wishes to know the results, including any unexpected news pertaining to him (Italian Data Protection Authority, 2018). In case of genetic tests or screening aimed at family reunification, the individuals involved must give their consent. They must also indicate whether they would like to be informed of the examination’s results, including any unexpected information, if doing so would directly and concretely benefit them in terms of treatment, prevention, or reproductive choice awareness (Italian Data Protection Authority, 2018). In general, genetic data must be disclosed directly to the individual in question or to others only through a written proxy provided by the individual, adopting all suitable means to prevent unauthorized knowledge by even co-participating data subjects. The communication in the hands of a proxy of the interested party shall be made in a closed envelope (Italian Data Protection Authority, 2018; Italian Data Protection Authority, 2016). The results of genetic testing (Casella et al., 2020; Fedeli et al., 2019) and screening, as well as the results of research, if they entail a concrete and direct benefit in terms of therapy, prevention or awareness of reproductive choices, including for those belonging to the same genetic line as the data subject, may be disclosed to the latter, at their request, if the data subject has expressly consented to this or such results are indispensable to prevent harm to their health, including reproductive risk, and if the data subject’s consent is not given or cannot be given due to actual unavailability. Although it is advised that consumers seek medical advice before acting on direct-to-consumer (DTC) genetic information, some consumers choose not to disclose their results to their healthcare providers, which raises serious bioethical questions regarding patient autonomy and mutations in the patient-doctor relationship (Majumder et al., 2021). A novel type of genetic consumerism is developing that may result in overdiagnosis, information seeking, or prediction, outside of conventional treatment settings. The role of the doctor in managing incidental findings in various family structures and striking a balance between autonomy and familial interests goes hand in hand with the necessity to always give comprehensive and straightforward information on the informative scope of genetic testing and the possible effects it may have on family dynamics. Only by respecting the patient’s autonomy and utilizing a multidisciplinary strategy that involves the expertise of multiple professionals can these ethical dilemmas be resolved. Both clinical and judicial settings may be challenged by several ethical issues such as respect for patient’s confidentiality and the potential duty to warn other relatives in case of inherited diseases. There could be significant bioethical tension. For example, it is prohibited to disclose a hereditary neurological syndrome to other family members in absence of a specific authorization from the patient itself. Informed consent, fairness, justice, autonomy, professional secrecy may be in bioethical tension with respect to physician activity and beliefs. Some cases provide significant bioethical insights and serve as iconic examples of the mentioned concepts. Consider the real case of a lady who has genetically based medullary thyroid cancer and who does not have enough information from her physician to fully understand that the mutation is inherited and that her children may also get the disease. Her daughter discovers years later that she has medullary thyroid carcinoma that has progressed. From an ethical perspective, one might be inclined to agree with those who believe that physicians have a duty to inform patients’ relatives about the genetically transmissible nature of the illness they are treating- even though this is an instance of a lack of information at the start of the story. The goal of the standard of care is to benefit the complete genetic family that is included in the domain of predicted risk. However, as mentioned above, the regulatory framework limits this ethical concern. Furthermore, the Court’s ruling in this particular case is representative. Although it is acknowledged that the doctor’s duty of care extends to third parties by warning them about a condition’s genetic transmissibility, this duty is deemed to be fulfilled by alerting the patient, and the duty of protection towards third parties is satisfied by effective communication with the latter.

### 4.2 Innovation

The study outlines as the real challenge in genetic testing field appears to be transforming the potential of genetic data from a personal, ultrapersonal dimension to a solidaristic one. The novel viewpoint is that of clinical utility of genetic data, which lies beyond the horizon of personalized and predictive medicine and uncovers a new dimension of universality. Health professionals must be aware of the importance of timing and specific *éxpertise* in genetic test communication, through a deep knowledge of the context in which they operate.

## 5 Conclusion

A focus on consent and confidentiality is indicated by the numerous works that have emerged from the human genome project and the general ideas around predictive medicine. In spite of the need for prudence and increased regulatory focus in this area, the Italian discipline seems to be adequately transparent and a guarantor of the principles of protecting patient anonymity. Major subgroups include consent and confidentiality, categories that also apply to the judicial use of genetic tests.

## References

[ref1] Ascencio-Carbajal T. Saruwatari-Zavala G. Navarro-Garcia F. Frixione E. (2021). Genetic/genomic testing: defining the parameters for ethical, legal and social implications (ELSI). BMC Med. Ethics 22:156. doi: 10.1186/s12910-021-00720-5, 34814901 PMC8609860

[ref2] Capasso E. Casella C. Marisei M. Tortora M. Briganti F. Di Lorenzo P. (2024). Imaging biobanks: operational limits, medical-legal and ethical reflections. Front. Digit. Health 6:1408619. doi: 10.3389/fdgth.2024.1408619, 39268200 PMC11391398

[ref3] Casella C. Carbone L. Conforti A. Marrone V. Cioffi G. Buonfantino C. . (2020). Preimplantation genetic testing: comparative analysis of jurisprudential regulations. Ital. J. Gynaecol. Obstet. 32:237. doi: 10.36129/jog.32.04.03

[ref4] Chou W. H. Chalker C. Sokolova A. O. Isharwal S. (2025). Prostate cancer and genetic contributions. Andrology 13, 1149–1157. doi: 10.1111/andr.13812, 39611376 PMC12119970

[ref5] Council of Europe (1988). Protocol to the Convention for the Protection of Human Rights and Fundamental Freedoms (European convention on human rights) as Amended by Protocol no. 11 Council of Europe Treaty Series 155

[ref6] European Union Charter of fundamental rights of the European Union, 2012/C 326/02 (2007). Available online at: https://www.refworld.org/legal/agreements/eu/2007/en/13901 (Accessed January 23, 2025).

[ref7] Fedeli P. Casella C. Buccelli C. Cannovo N. Graziano V. (2019). Genetic research: the role of citizens, public health and international stakeholders. Open Public Health J. 12, 106–113. doi: 10.2174/1874944501912010106

[ref8] Federazione Nazionale degli Ordini dei Medici Chirurghi e degli Odontoiatri. Italian Code of Medical Ethics (2014). Available online at: https://portale.fnomceo.it/codice-deontologico/ (Accessed July 30, 2025).

[ref9] Giefing M. Wierzbicka M. Szyfter K. Brenner J. C. Braakhuis B. J. Brakenhoff R. H. . (2016). Moving towards personalised therapy in head and neck squamous cell carcinoma through analysis of next generation sequencing data. Eur. J. Cancer 55, 147–157. doi: 10.1016/j.ejca.2015.10.070, 26851381 PMC4761501

[ref10] Hamilton J. G. Abdiwahab E. Edwards H. M. Fang M. L. Jdayani A. Breslau E. S. (2017). Primary care providers' cancer genetic testing-related knowledge, attitudes, and communication behaviors: a systematic review and research agenda. J. Gen. Intern. Med. 32, 315–324. doi: 10.1007/s11606-016-3943-4, 27995427 PMC5331015

[ref11] Italian Data Protection Authority (2016). Authorisation no. 8/2016 - general authorisation to process genetic data. Register of provisions No. 530 of December 15, 2016.

[ref12] Italian Data Protection Authority (2018). Provision containing the requirements for the processing of special categories of data, pursuant to article 21, paragraph 1 of legislative decree no. 101 of august 10, 2018. Register of provisions no. 146 of June 5, 2019. Annex 1, section 4: requirements regarding the processing of genetic data.

[ref13] Law 22 December 2017, no. 219 (2018). Provisions for Informed Consent and Advance Treatment Directives. Gazzetta Ufficiale della Repubblica Italiana.

[ref14] Law 7 December 2000, No. 397 (2001). Provisions on Defensive Investigations. Gazzetta Ufficiale della Repubblica Italiana.

[ref15] legislation.gov.uk (2016). Regulation (EU) 2016/679 of the European Parliament and of the council of 27 April 2016, on the protection of natural persons with regard to the processing of personal data and on the free movement of such data and repealing directive 95/46/EC (general data protection regulation).

[ref16] Lolkema M. P. Gadellaa-van Hooijdonk C. G. Bredenoord A. L. Kapitein P. Roach N. Cuppen E. . (2013). Ethical, legal, and counseling challenges surrounding the return of genetic results in oncology. J. Clin. Oncol. 31, 1842–1848. doi: 10.1200/JCO.2012.45.2789 23589552

[ref17] Majumder M. A. Guerrini C. J. McGuire A. L. (2021). Direct-to-consumer genetic testing: value and risk. Annu. Rev. Med. 72, 151–166. doi: 10.1146/annurev-med-070119-114727 32735764

[ref18] National Bioethics Committee. (2016). Opinion on the management of “incidental findings” in genomic investigations with new technology platforms, March 17. Available online at: https://bioetica.governo.it/media/4369/p123_2016_incidental-findings_en.pdf, (Accessed October 6, 2025).

[ref19] Nufeld Council on Bioethics. Genetic Screening Ethical Issues (1993).

[ref20] Peterson E. B. Chou W. S. Gaysynsky A. Krakow M. Elrick A. Khoury M. J. . (2018). Communication of cancer-related genetic and genomic information: a landscape analysis of reviews. Transl. Behav. Med. 8, 59–70. doi: 10.1093/tbm/ibx063, 29385592 PMC6065548

[ref21] Schneider S. A. Schneider U. H. (2024). Rights and duties of genetic counsellors in Germany related to relatives at risk: comparative thoughts on the German genetic diagnostics act. J. Med. Ethics 50, 324–331. doi: 10.1136/jme-2023-108975 37451858

[ref22] The Nuremberg Code (1946). Available online at: https://media.tghn.org/medialibrary/2011/04/BMJ_No_7070_Volume_313_The_Nuremberg_Code.pdf (Accessed July 30, 2025).

[ref23] UNESCO. (1997). The Universal Declaration on the Human Genome and Human rights. Available online at: https://www.unesco.org/en/legal-affairs/universal-declaration-human-genome-and-human-rights?hub=387 (Accessed October 6, 2025).

[ref24] United Nations Educational Scientific and Cultural Organization (2005). Universal Declaration on Bioethics and Human Rights.

[ref25] United States National Commission for the Protection of Human Subjects of Biomedical and Behavioral Research (1978). The Belmont Report: Ethical Principles and Guidelines for the Protection of human Subjects of Research. DHEW Publication no (OS) 780012. Bethesda: The Commission; for sale by the Supt. of Docs., U.S. Govt. Print. Off.

[ref26] World Health Organization. Advisory Committee on Health Research (2002). Genomics and World Health Report of the Advisory Committee on Health Research. Geneva: World Health Organization.

[ref27] World Medical Association (1964). WMA Declaration of Helsinki – Ethical Principles for Medical Research Involving Human Subjects. 19886379

[ref28] World Medical Association (1981). WMA Declaration of Lisbon on the Rights of the Patient.

